# Top 10 Histological Mimics of Neuroendocrine Carcinoma You Should Not Miss in the Head and Neck

**DOI:** 10.1007/s12105-022-01521-x

**Published:** 2023-03-20

**Authors:** C. Christofer Juhlin, Munita Bal

**Affiliations:** 1 Department of Oncology-Pathology, BioClinicum J6:20, Karolinska Institutet, Solna, 171 64 Stockholm, Sweden; 2 Department of Pathology and Cancer Diagnostics, Karolinska University Hospital, Stockholm, Sweden; 3 Department of Pathology, Tata Memorial Centre, Homi Bhabha National Institute, Mumbai, 400012 India

**Keywords:** Neuroendocrine carcinoma, Head and neck, Differential, Immunohistochemistry, Review

## Abstract

**Background:**

The spectrum of neuroendocrine neoplasia (NEN) of the head and neck region is wide-ranging and diverse, including a variety of diagnoses stretching from benign and low-malignant tumor forms to highly proliferative, poor prognosis neuroendocrine carcinoma (NEC). Moreover, there are several non-neuroendocrine differential diagnoses to keep in mind as well, displaying various degree of morphological and/or immunohistochemical overlap with *bona fide* neuroendocrine lesions.

**Methods:**

Review.

**Results:**

While the growth patterns may vary, well-differentiated NEN usually display a stippled “salt and pepper” chromatin, a granular cytoplasm, and unequivocal expression of neuroendocrine markers such as chromogranin A and synaptophysin. However, these features are often less pronounced in NEC, which may cause diagnostic confusion—not the least since several non-NEC head and neck tumors may exhibit morphological similarities and focal neuroendocrine differentiation.

**Conclusion:**

As patients with NEC may require specific adjuvant treatment and follow-up, knowledge regarding differential diagnoses and potential pitfalls is therefore clinically relevant. In this review, the top ten morphological and/or immunohistochemical mimics of NEC are detailed in terms of histology, immunohistochemistry, and molecular genetics.

## Introduction

Neuroendocrine neoplasia (NEN) of the head and neck region are spectacular lesions, often accompanied by unique morphological, immunohistochemical, hormonal, and/or genetic features. While most of these lesions mainly occur sporadically in adult patients, subsets are intimately coupled to genetically inherit syndromic disease and may therefore present in younger patients [1, 2]. In addition to the demographic diversity, neuroendocrine tumors (NETs) may be notoriously difficult to prognosticate—as the metastatic potential may be challenging to assess by morphology alone. Consequently, pathologists have developed risk stratification algorithms for various NENs in order to assess the risk of disease progression, with the proliferation index (as estimated by mitotic index and/or the Ki-67 labeling index) proving particularly important [3–5]. Indeed, these parameters are nowadays routinely used in grading NENs, with neuroendocrine carcinoma (NEC) displaying the highest proliferation index [3]. Due to the highly proliferative nature of this entity, distant metastases and death due to disease are common outcomes for the NEC patient category [6]. Therefore, it is imperative to correctly identify these lesions in a timely fashion, and care must be taken to not confuse NEC with malignant, non-NEC neoplasms with focal immunoreactivity to neuroendocrine markers, as well as the clinically more indolent well-differentiated NETs.

## Neuroendocrine Carcinoma of the Head and Neck Region

**Fig. 1:**
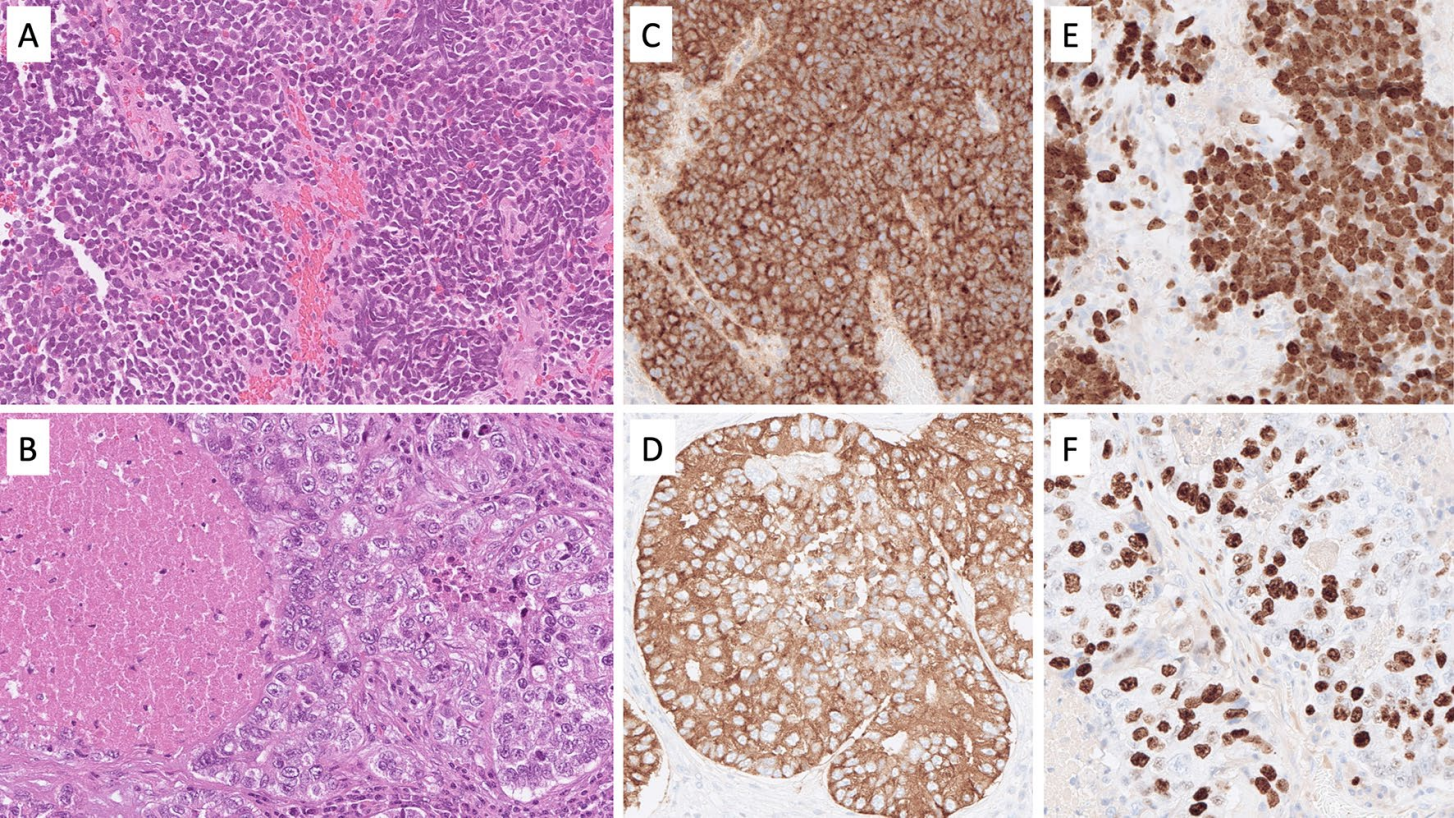
Neuroendocrine carcinoma of the head and neck region. ** A** Sinonasal small cell neuroendocrine carcinoma exhibiting solid sheets of tumor cells with high nuclear/cytoplasmic ratio and nuclear molding. **B** Salivary gland large cell neuroendocrine carcinoma with nuclear pleomorphism and geographic tumor cell necrosis. **C**, **D** Immunohistochemistry for synaptophysin and chromogranin A usually reveal diffusely positivity. **E**, **F** The Ki-67 proliferation index is always above 20%, here exemplified by 90% and 70%, respectively

The most clinically urgent NEN subtype is NEC, a poorly differentiated, highly proliferative, malignant tumor of poor prognosis exhibiting significant tumor necrosis and destructively invasive features [3]. In the head and neck region, NEC may develop within the paranasal sinuses, the nasal cavity, the oro- and hypopharynx, the salivary glands, the oral cavity, and the larynx [7–11]. NEC of the head and neck region is further divided into small cell neuroendocrine carcinoma (SCNEC), large cell neuroendocrine carcinoma (LCNEC), and mixed NEC with non-neuroendocrine neoplasms [8]. While SCNEC is composed of diffusely arranged small cells with a high nuclear/cytoplasmic ratio, nuclear molding, and necrosis (Fig. 1A), LCNEC usually exhibits hyperchromatic and pleomorphic tumor cell nuclei (sometimes palisading) with prominent nucleoli (Fig. 1B) arranged in nests, sheets, or trabeculae. Necrosis is often widespread. Immunohistochemistry (IHC) is usually positive for neuroendocrine markers, such as chromogranin A and synaptophysin (Fig. 1C, D), but expression of both markers may be absent in some lesions [12]. Inclusion of second-generation neuroendocrine markers, such as INSM1, may be of value in these instances [13]. Both SCNEC and LCNEC express keratins, and the expression may be faint and dot like in the former entity. This feature can be particularly helpful in distinguishing from paragangliomas. The Ki-67 proliferation index is usually high, always > 20%, and frequently between 55 and 100% (Fig. 1E, F). Subsets of oropharyngeal NECs may be human papillomavirus (HPV)-driven neoplasms, exhibiting strong p16 immunoreactivity [14]. Most head and neck NECs are positive for p53 by immunohistochemistry and negative for retinoblastoma protein (pRb), which is due to frequent somatic mutations in the tumor suppressors *TP53* and *RB1* [15]. Interestingly, subsets of cases also harbor pathogenic variants in potentially actionable therapeutic target genes associated with the NOTCH and PI3K/AKT/mTOR pathways [15].

## Head and Neck Mimics of Neuroendocrine Carcinoma

**Fig. 2:**
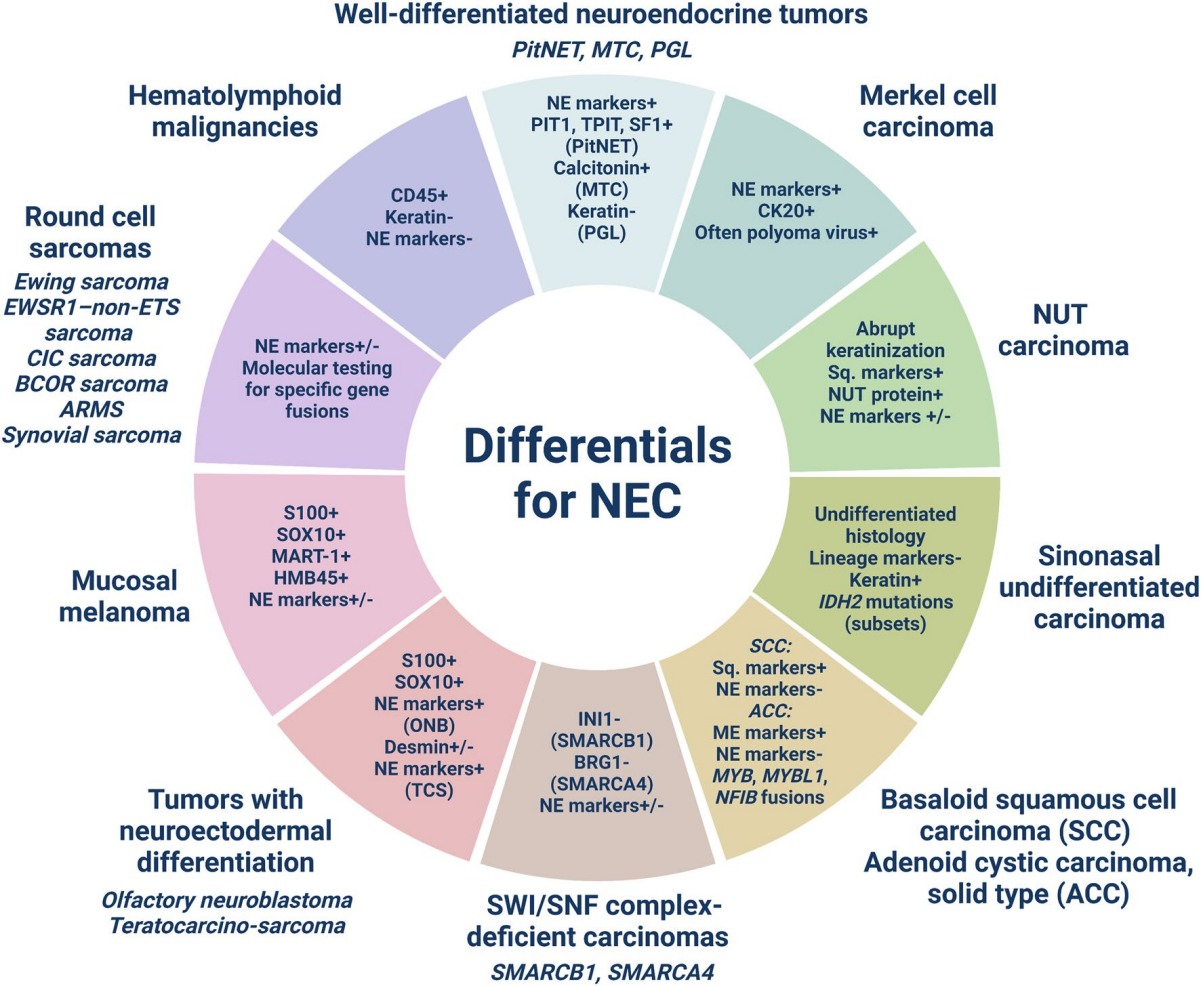
Schematic overview of the top ten differential diagnoses of neuroendocrine carcinoma (NEC) in the head and neck region with key immunohistochemical and molecular attributes. *PitNET* pituitary neuroendocrine tumor, *MTC* medullary thyroid carcinoma, *PGL* paraganglioma, *NE* neuroendocrine, *ME* myoepithelial, *Sq* squamous, *SCC* basaloid squamous cell carcinoma, *ACC* adenoid cystic carcinoma, *ONB* olfactory neuroblastoma, *TCS* teratocarcinosarcoma, *ARMS* alveolar rhabdomyosarcoma. Created using BioRender.com

A plethora of entities of diverse lineages must be excluded when considering a diagnosis of NEC. On one hand, the cellular, closely packed monotonous tumor cells in SCNEC bring nearly all small round blue cell tumors into the differential diagnoses, while on the other hand, miscellaneous head and neck epithelial malignancies remain close mimics of LCNEC. The challenges are particularly heightened when dealing with small tissue volumes in biopsy material. Awareness of the histomorphologic spectrum, immunophenotypic nuances, and molecular traits of the diverse mimics and employing a stepwise algorithmic approach can aid in reaching an accurate diagnosis (Fig. 2, Table 1). Given the possible differential diagnostic dilemmas discussed above, we provide a review of the top ten morphological and immunohistochemical NEC mimics that the practicing pathologist should not miss.

**Table 1:** Immunohistochemistry in differential diagnoses of small cell neuroendocrine carcinoma

Marker	NEC	WDNET	BSCC	ACC solid	SNUC	SMARCB1	SMARCA4	ONB	TCS	Melanoma	NUT	Lymphoma	MCA	ES	RMS	PDSS
Keratin	+	+	+ +	+	+	+	+	−r	+ e	−	+	−	+	−/ +	−/ +	+
SYP	+	+ +	−	−	−/ +	−/ +	±	+	+ n	−/ +	−	−	+	±	−/ +	−
CgA	+	+ +	−	−	−/ +	−/ +	±	+	+ n	−	−	−	+	−	−/ +	−
INSM1	+	+ +	−	−	−	−/ +	±	+	+ n	− r	−	−	+	±	±	−
CD56	+	+ +	−	−	−	−	+	+	− n	−/ +	−	−	+	−	+	−
NKX2.2	±	±	−	−	−	−	−	±	±	−/ +	−	−	−/ +	+	−	−
p40	−/ + f	−	+ +	+	−	+	−	−	+ f	−	+	−	−	−	−	−
CD117	±	−	±	+	−	−	−	±	−	±	−	−	−	−	−	−
MYB	−	−	−	+	−	−	−	−	−	−	−	−	−	−	−	−
S100/ SOX10	−	−	±	+	−	−	−	+	−	+	−	−	−	−	−	−/ +
Melan A/ HMB45	−	−	−	−	−	−	−	−	−	+	−	−	−	−	−	−
LCA	−	−	−	−	−	−	−	−	−	−	−	+	−	−	−	−
CK20	−	−	−	−	−	−	−	−	−	−	−	−	+	−	−	−
Polyoma virus	−	−	−	−	−	−	−	−	−	−	−	−	+	−	−	−
p16	** + **	−	−	+ f	±	±	−	−	−	−/ +	−	−	−	−	−	−
NUT	**−**	−	−	−	−	−	−	−	−	−	+	−	−	−	−	−
Desmin	−	−	−	−	−	−	−	−	± s	−	−	−	−	−	+	**−**
SMA	−	−	−	−	−	−	−	−	−	−	−	−	−	−	−	−
CD99	−/ +	−	−	−	−	−	−	−	±	−	−	−	−/ +	+	± cy	+
Fli1	−	−	−	−	−	−	−	−	−	−	−	−	−/ +	+	−	−
TTF1	** + **	−	−	−	−	−	−	−	−	−	−	−	−	−	−	−
SMARCB1	+	+	+	+	+	−	+	+	+	+	+	+	+	+	+	+ f*
SMARCA4	+	+	+	+	+	+	−	+	−	+	+	+	+	+	+	+
IDH1/2	−	−	−	−	± #	−	−	−	−	−	−	−	−	−	−	−
TLE1	−r	−/ +	+/ −	−	−	−	−	−	−	+/ −	−	−	−r	−/ +	−/ +	+ +
*SS18::SSX*	−	−	−	−	−	−	−	−	−	−	−	−	−	−	−	+

*NEC* Neuroendocrine carcinoma; *WDNET* Well-differentiated neuroendocrine tumor; *BSCC* Basaloid squamous cell carcinoma; *ACC* Adenoid cystic carcinoma; *SNUC* Sinonasal undifferentiated carcinoma; *SMARCB1* SMARCB1 (INI1) deficient carcinoma; *SMARCA4* SMARCA4 (BRG1) deficient carcinoma; *ONB* Olfactory Neuroblastoma; TCS; *NUT* NUT carcinoma; *MCA* Merkel cell carcinoma; *ES* Ewing sarcoma; *RMS* Rhabdomyosarcoma; *PDSS* Poorly differentiated synovial sarcoma; *SYP* synaptophysin; *CgA* chromogranin A

−/+: more frequently negative than positive

±: more frequently positive than negative

++: strong and diffuse positive

r: rare cases show focal expression

f: focal

e: in the epithelial component

n: in the neuroectodermal component

s: in the rhabdomyosarcomatous component, if present

cy: cytoplasmic

f*: mosaic pattern of loss

#: in IDH-mutant subset

### Well-Differentiated Neuroendocrine Tumors

Well-differentiated NETs (WDNETs) encompass well-differentiated neoplasms with neuroendocrine features recognizable on light microscopy, i.e., exhibiting cellular monotony, random anisonucleosis, stippled nuclear chromatin, and varied growth patterns (nests, trabeculae, cords, festoons, rosettes). These tumors are strongly immunoreactive with neuroendocrine markers. WDNETs and NECs are clinically and genetically distinct entities with divergent treatments and outcomes. Therefore, a clear distinction between WDNET and NEC is necessary. This is usually not problematic, however, tends to be challenging in cases with limited or crushed tissue.

#### Pituitary Neuroendocrine Tumor (PitNET)

**Fig. 3:**
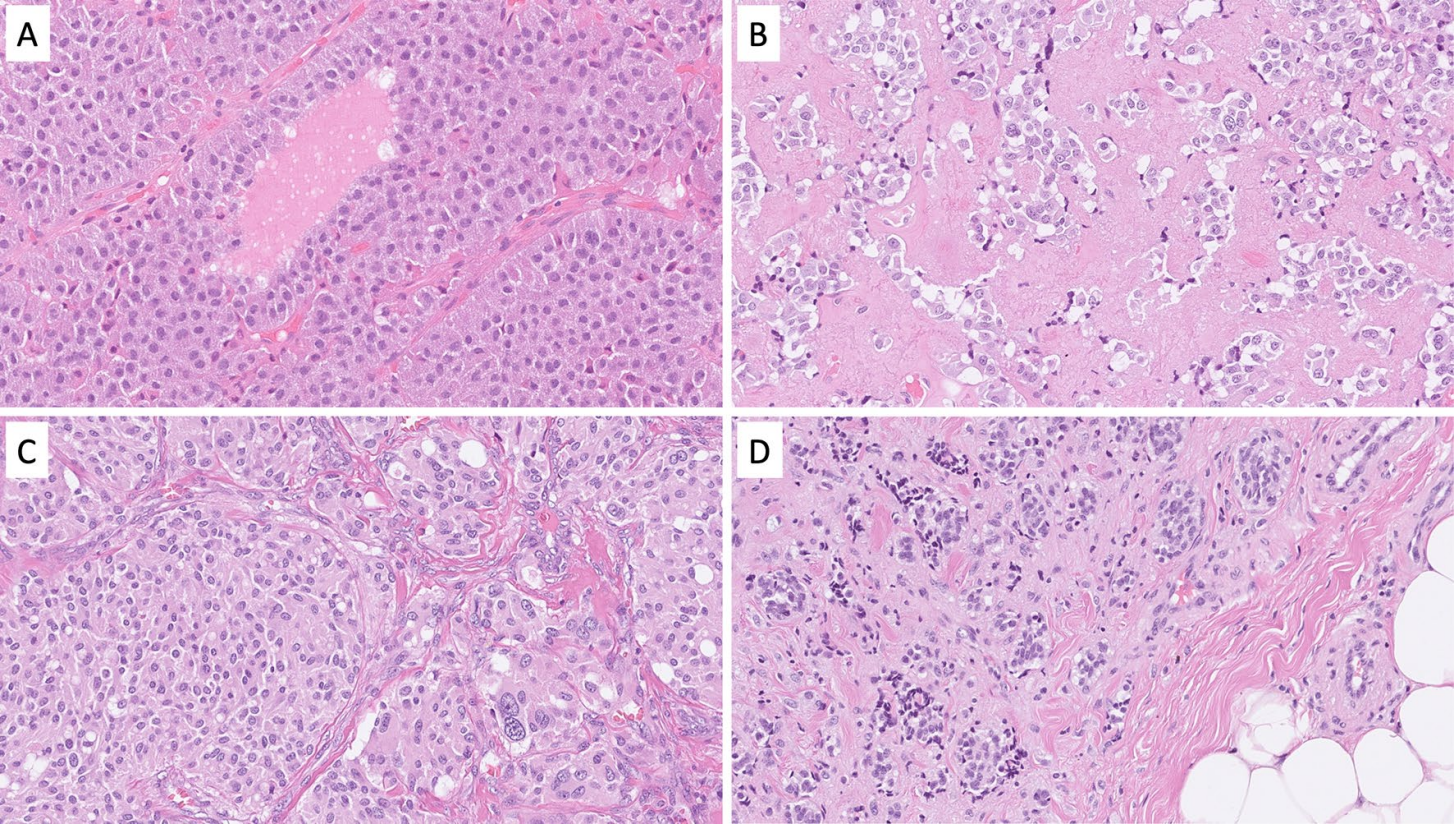
Differential diagnosis of a neuroendocrine carcinoma: Morphological attributes of different well-differentiated neuroendocrine tumors of the head and neck region. **A** Somatotroph pituitary neuroendocrine tumor (PitNET) characterized by large, acidophilic and granular cells with little nuclear pleomorphism. **B** Medullary thyroid carcinoma comes in different forms and shapes, but regularly display cells with amphophilic to basophilic cytoplasm and neuroendocrine-type chromatin and often grow in an amyloid background. **C** Carotid body paraganglioma exhibiting a nested appearance and cells with abundant, granular cytoplasm. Nuclear pleomorphism may be present but correlate poorly to metastatic behavior. **D** Neuroendocrine neoplasia of unknown primary (NEN-UP) metastatic to the skin. This lesion required extensive immunohistochemistry and finally led to a diagnosis of a primary pulmonary carcinoid

Pituitary neuroendocrine tumors (PitNETs) are well-differentiated adenohypophyseal lesions that may cause a wide variety of symptoms depending on its specific hormone production. PitNETs may be encountered in the sinonasal tract, most frequently in the sphenoid sinus, due to invasion from a sellar tumor or rarely as ectopic PitNET. The previous terminology “pituitary adenoma” and “pituitary carcinoma” are no longer recommended [16]. PitNETs should be subtyped in terms of tumor cell lineage and expression of pituitary hormones by the use of IHC, and tumors are derived from a PIT1 lineage (i.e., somatotroph, lactotroph, and thyrotroph), a TPIT lineage (corticotroph tumors), an SF1 lineage (gonadotroph tumors), or tumors without a distinct lineage (plurihormonal lesions and hormonally silent “null cell tumors”) [16]. Most PitNETs are characterized by a hypercellular and well-differentiated mass composed of monomorphic tumor cells (Fig. 3A). Although there are morphological clues regarding the PitNET subtype, the distinction is not entirely reliable without IHC and therefore all PitNETs should be assessed for PIT1, TPIT, and SF1 immunoreactivity [16]. Hormone stains are also recommended, including growth hormone (GH), prolactin (PRL), and beta-thyroid-stimulating hormone (β-TSH) for PIT1 lineage tumors; adrenocorticotropic hormone (ACTH) for TPIT lineage tumors; and beta-follicle-stimulating hormone (β-FSH) and beta-luteinizing hormone (β-LH) for SF1 lineage tumors [17]. Keratin IHC can also be useful (CAM5.2, AE1/AE3, and/or CK18) to subtype somatotroph PitNETs as either densely or sparsely granulated, characterized by a perinuclear or globular cytoplasmic stain, respectively [16]. Metastatic PitNETs are rarely encountered, and when metastatic, most commonly affect liver, bone, lung, and lymph nodes [18].

#### Medullary Thyroid Carcinoma (MTC)

Medullary thyroid carcinoma (MTC) is a neuroendocrine tumor derived from the calcitonin-producing C cells of the thyroid gland. Although the majority of tumors arise sporadically, up to 25% are thought to be associated with multiple endocrine neoplasia type 2 (MEN2A or MEN2B) syndrome, with the affected patient demonstrating activating, constitutional pathogenic variants in the *RET* proto-oncogene [2, 19]. Sporadic tumors are usually driven by somatic *RET* mutations or mutually exclusive *RAS* gene mutations [20]. There are numerous morphological MTC patterns reported, but they are not routinely classified on a histological basis as there is no established correlation to either genotype or clinical outcomes [19]. MTCs are characterized by round (sometimes plasmacytoid, polygonal, or spindle shaped) cells in nests with an interdigitating stroma exhibiting various amounts of amyloid deposition (Fig. 3B). The cytoplasm is usually amphophilic and granular due to their secretory content. MTCs routinely express neuroendocrine markers as well as signs of thyroid differentiation (TTF1), whereas monoclonal PAX8 expression is lacking, as is thyroglobulin (the latter a consequence of the non-follicular cell origin). The hallmark of MTCs is calcitonin immunoreactivity, although the stain can vary in intensity and spatial distribution. In the metastatic setting without an established thyroid lesion, care must be taken not to prematurely assume that a neuroendocrine tumor with focal calcitonin and TTF1 expression is a metastatic MTC—as cases of laryngeal NETs with aberrant expression of these markers have been reported [21]. In terms of prognosis, the 2022 World Health Organization (WHO) classification of endocrine and neuroendocrine tumors recommends that MTCs be graded based on the mitotic index, the presence of tumor necrosis, and the Ki-67 proliferation index, in which high-grade lesions display necrosis, a mitotic count ≥ 5 per 2 mm^2^, and/or a Ki-67 index of ≥ 5% [5, 19]. In terms of metastatic disease, most MTCs spread regionally to neck lymph nodes, but subsets of cases may also spread to the liver, lungs, and bone. It may be worth noting that rare cases of MTC metastatic to the parotid and pituitary glands have been reported, which potentially could constitute differential diagnostic conundrums [22, 23]. Moreover, subsets of MTCs may display a small cell phenotype, further complicating the histological work-up if NEC is suspected [23].

#### Paraganglioma (PGL)

Head and neck paragangliomas (PGLs) are usually parasympathetic, non-functioning neuroendocrine tumors, which sets them apart from their infra-diaphragmatic, norepinephrine, and/or epinephrine-producing counterparts [1]. They are collectively the most inheritable of all human neoplasia with approximately 40% of patients carrying an underlying constitutional genetic event, while the metastatic potential of these lesions is usually low [24]. Patients with constitutional pathogenic variants in *succinate dehydrogenase (SDH) subunit A*, *B*, *C*, *D*, or *AF2* (*SDHA, SDHB, SDHC, SDHD, SDHAF2*) harbor an increased risk of developing head and neck paraganglioma with a low risk of disseminated disease [25]. The underlying molecular biology is complex, with tumors showing a higher risk of metastases often driven by mutations in tricarboxylic acid (TCA) cycle genes (not only restricted to *SDH* genes) that will lead to TCA cycle arrest and accumulation of early metabolites, which in turn may activate oncogenic hypoxia-inducible factor (HIF) pathways [26]. However, subsets of cases are driven by mutations in various kinase-associated pathways, and these lesions usually tend to be non-metastatic. Therefore, there is a well-developed genotype–phenotype correlation which can be assessed by histopathology and immunohistochemistry: a positive SDHB immunostain strongly argues against mutational inactivation of either *SDHB*, *SDHC*, or *SDHD* genes—in turn arguing against (although not excluding) the risk of metastatic potential [27, 28]. When presenting in characteristic locations, such as the carotid bifurcation or the jugulotympanic area, a head and neck paraganglioma is quite easily distinguished by morphological assessment. The tumor cells are usually round to oval and arranged in small nests (so-called “zellballen”) embedded in a highly vascular stroma (Fig. 3C). The cytoplasm is granular with an amphophilic or basophilic appearance, and nuclear pleomorphism is usually limited to absent. Mitoses and tumor necrosis are rarely detected. Tumor cells are positive for chromogranin A, synaptophysin, and GATA3, while consistently keratin negative [29]. An S100 protein (or SOX10) immunohistochemistry identifies the sustentacular network of cells supporting the tumor cells—but the finding of sustentacular cells is not diagnostic for paraganglioma, as other neuroendocrine tumors also may exhibit this feature [30]. Using functional IHC, most head and neck paragangliomas are positive for choline acetyltransferase, an enzyme in the acetylcholine biosynthesis pathway, while often negative for enzymes responsible for catecholamine production, such as tyrosine hydroxylase [31, 32]. Metastatic head and neck paraganglioma usually spread to regional lymph nodes, while distant site involvement is rare [33].

#### Metastastic Neuroendocrine Neoplasia of Unknown Primary

NENs of unknown primary (NEN-UPs) are metastatic lesions without a known primary tumor location, a finding reported in 12–22% of NEN patients [34]. The importance of identifying the primary site cannot be underestimated given that the various clinical and prognostic features of NENs depend on the tumor origin site. There are several morphological clues that can be used to properly identity a NEN-UP, including amyloid deposits in MTC, psammoma bodies in somatostatinoma, a hyalinized stroma in insulinoma, as well as the hyaline globules and basophilic cytoplasm of pheochromocytoma [34]. Even so, it is not unusual for a metastatic NEN-UP to be characterized by nested cells with little or no morphological findings unique to the primary tumor site (Fig. 3D). From an immunohistochemical perspective, various combinations of neuroendocrine marker and transcription factors results may be useful. For example, TTF1 may help identify pulmonary carcinoids and MTC, PDX1 may assist in recognizing NENs of the upper gastrointestinal tract including the pancreas, whereas CDX2 and SATB2 may highlight NENs of the lower gastrointestinal tract. In addition, testing for various hormones may be useful, including calcitonin for MTC, serotonin for lower gastrointestinal NENs, islet hormones for pancreatic NENs, and GLP1 for rectal NENs, to name just a few [35, 36]. NECs outside of the head and neck area occasionally may metastasize to the jaws and major salivary glands [36]. Indeed, metastatic neuroendocrine tumors to the parotid gland accounted for 22% of all metastatic tumors to this organ in a recent case series, and most cases were either pulmonary NECs, Merkel cell carcinomas (MCCs), or MTCs [37]. If not previously known, a hypothetical IHC panel for NEN-UPs metastatic to the salivary glands would therefore need at least TTF1, calcitonin, and CK20, in addition to neuroendocrine markers and Ki-67.

### Merkel Cell Carcinoma

**Fig. 4:**
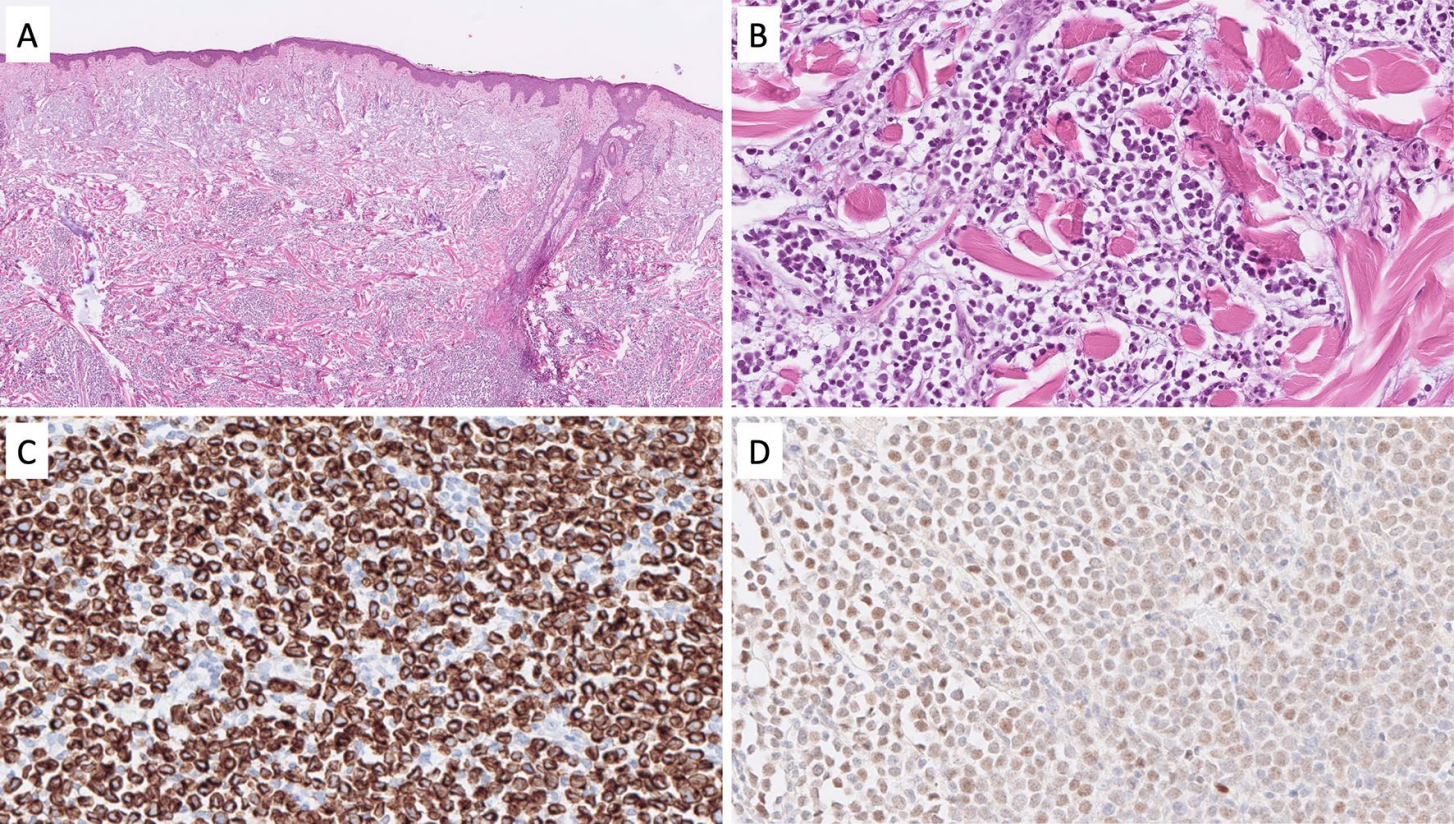
Differential diagnosis of a neuroendocrine carcinoma: Merkel cell carcinoma (MCC). **A**, **B** MCC characterized by a diffusely infiltrative tumor within the dermis and subcutaneous space growing in sheets and trabeculae. Cells exhibit a high nuclear-to-cytoplasmic ratio, and mitoses and apoptotic bodies are plentiful. **C** Cytokeratin 20 (CK20) usually present with a perinuclear or dot-like pattern. **D** The majority of MCCs are positive for the Merkel cell polyoma virus antigen

Merkel cell carcinoma (MCC) is a rare neuroendocrine carcinoma of the skin with an estimated incidence of 2.2 cases per million person-years, afflicting predominantly older patients [38]. The tumors are either driven by UV-induced mutations or by a Merkel cell polyoma virus infection, and the exact proportion of these etiologies varies with geographic distribution [39]. From a morphological perspective, MCCs are composed of solid arrangements of monomorphic tumor cells with a high nuclear/cytoplasmic ratio, smudged nuclear chromatin, indistinct nucleoli, and displaying innumerable mitoses (Fig. 4A, B). The IHC profile is characteristically neuroendocrine [40], while a perinuclear, “dot-like” keratin stain (most strikingly with CK20) is characteristic of MCC (Fig. 4C). The Ki-67 proliferation index is usually exceedingly high (> 90%). Moreover, virus-driven MCCs are positive for the Merkel cell polyoma antigen (Fig. 4D). When presenting as a primary tumor, the diagnosis is usually quite straight-forward, but metastatic lesions may cause diagnostic difficulties if the primary tumor is not known. To complicate matters even more, subsets of MCCs have been reported to originate from mucosal linings of the upper respiratory and GI tracts and might be clinically silent [41].

### NUT Carcinoma

**Fig. 5:**
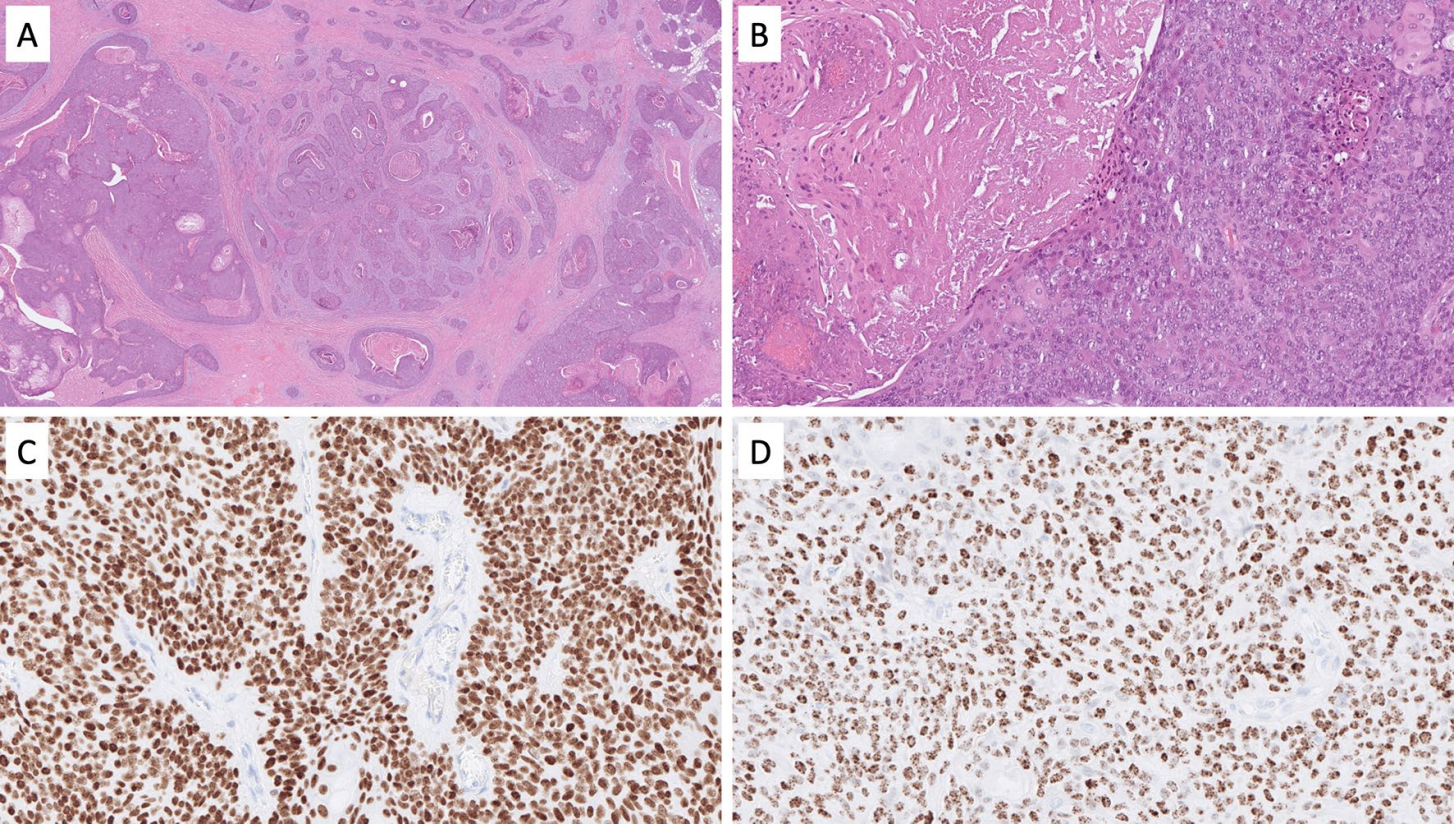
Differential diagnosis of a neuroendocrine carcinoma: NUT carcinoma. **A**, **B** Primitive cells arranged in solid sheets with areas of abrupt keratinization. **C** Immunohistochemical positivity is noted for squamous cell markers, such as p40. **D** Nuclear NUT protein expression is evident, indicating an underlying translocation involving the *NUT* gene

NUT carcinoma is a highly aggressive tumor primarily affecting young patients, often presenting in the midline of the thorax and head and neck regions [42, 43]. On the histological level, NUT carcinoma is composed of small to intermediate cells with an undifferentiated, primitive, and monotonous appearance (Fig. 5A). Mitotic figures and necrosis are easily identified. A significant subset exhibits abrupt keratinization (Fig. 5B). Using IHC, NUT carcinomas are epithelial neoplasms, reacting with keratins and squamous markers, such as CK5/6, p63, and p40 (Fig. 5C). CD34 is also positive in approximately 50% of cases [43]. NUT carcinoma is driven by *NUTM1* gene rearrangements [43], and NUT protein immunohistochemistry (Fig. 5D) is useful to highlight this genetic aberrancy—as NUT protein expression is not normally seen in cells outside of the testis and ciliary ganglion [44]. Interestingly, neuroendocrine differentiation has been reported, with a high level of suspicion required when considering NEC in young patients by incorporating NUT immunohistochemistry [45].

### Sinonasal Undifferentiated Carcinoma

Sinonasal undifferentiated carcinoma (SNUC) is a rare but highly aggressive epithelial neoplasia lacking morphological and immunohistochemical evidence of lineage (including glandular, squamous, neuroendocrine, or mesenchymal differentiation) [46]. Thus, it is a diagnosis of exclusion in which a broad range of possible differential diagnoses must be considered. Usually presenting as a large mass in the sinonasal tract, these tumors are often invasive at diagnosis [47]. SNUC exhibits high-grade histology with uniform tumor cells growing in sheets, lobules, nests or trabeculae, lacking squamous, or glandular differentiation. Tumor cells express keratins, while p40 is negative and p63 may exhibit weak and unspecific staining [48] (Fig. 2). Subsets of SNUC may express patchy chromogranin A and/or synaptophysin immunoreactivity, making them a potential differential diagnosis in the work-up of NEC of the head and neck region [49]. Somatic *IDH2* mutations have been identified in large subsets of SNUC and are readily identifiable using sequencing analysis, while IDH immunohistochemistry has proven inconsistent in pinpointing IDH2-mutated SNUC [50, 51].

### Basaloid Squamous Cell Carcinoma

**Fig. 6:**
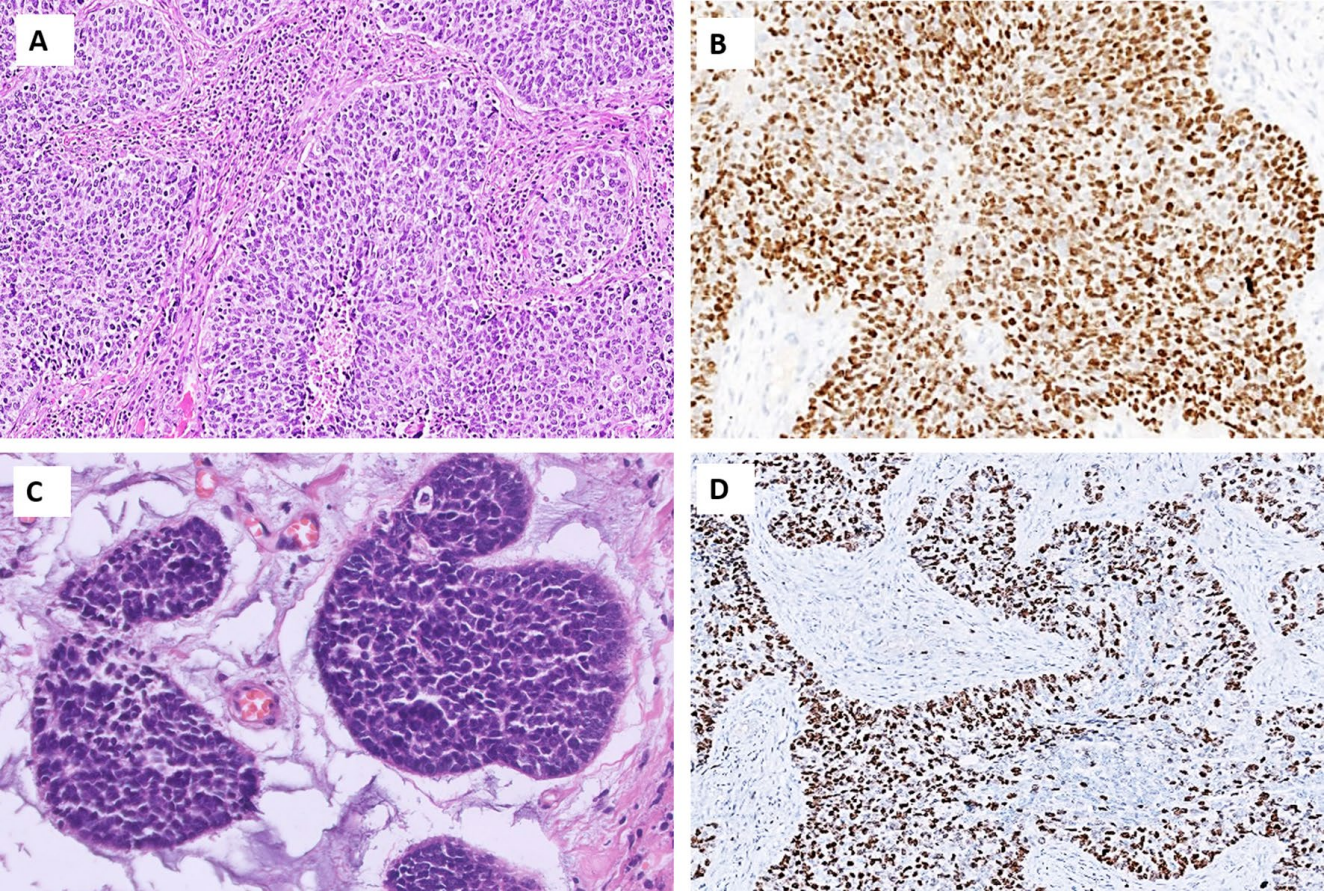
Differential diagnosis of a neuroendocrine carcinoma (NEC): Epithelial morphology. **A** Basaloid squamous carcinoma (BSC) with lobules of basaloid cells exhibiting peripheral palisading and central comedonecrosis. **B** Immunohistochemistry for p40 is diffusely and strongly positive in BSC. **C** Solid adenoid cystic carcinoma (ACC) is composed of solid nests and lobules of basaloid cells with hyperchromatic angulated nuclei and sparse ducts imparting a blue tumor appearance to the tumor. **D** Immunohistochemistry for myoepithelial marker SOX10 in ACC is helpful in distinguishing it from NEC

Squamous cell carcinoma (SCC) is the most common histological type of cancer in the head and neck region. While the diagnosis of a differentiated SCC is not problematic, the basaloid SCC subtype is a common differential diagnosis of SCNEC, particularly in biopsy material. Basaloid SCC remains an uncommon malignancy that is associated with aggressive clinical behavior and poor median survival (18 months) [52]. Closely packed basaloid cells and lack of significant keratinization typically impart a blue cell tumor appearance at low power that resembles SCNEC. Lobules, adenoid/pseudoglandular structures or variably anastomosing islands of tumor cells exhibiting peripheral palisading, thickened basement membrane-like material, and central comedonecrosis are typical histological features (Fig. 6A) [53]. The neoplastic cells show pleomorphic hyperchromatic nuclei with scanty cytoplasm. The presence of carcinoma in situ or areas of abrupt squamous differentiation (keratin pearl formation) are useful clues to the diagnosis. Mitoses are usually easily identified. There is usually strong and diffuse immunoreactivity for pancytokeratin, p40 (Fig. 6B), and p63, while neuroendocrine markers are negative. SOX10, CD117, and carcinoembryonic antigen (CEA) are notably positive in a subset of basaloid SCC [53–55], features not seen in SCNEC.

### Adenoid Cystic Carcinoma Solid Pattern

While histological features of conventional adenoid cystic carcinoma (ACC) (with its typical cribriform architecture, dual-layered tubules lined by epithelial–myoepithelial cells, and luminal basophilic matrix) are quite characteristic and easy to diagnose, ACC with a solid pattern can resemble SCNEC, especially in a limited or small biopsy. Solid ACC is composed of diffuse sheets of basaloid cells that are largely devoid of the hallmark cribriform glands or tubules (Fig. 6C). These tumors commonly have increased mitoses and tumor necrosis. However, distinction from SCNEC can be readily achieved with the use of selected IHC. ACC shows positivity for epithelial (CK7, CEA, EMA) and myoepithelial markers (S100 protein, SOX10, SMA, calponin) (Fig. 6D), while is negative for neuroendocrine markers. CD117 positivity may be seen in both ACC [56] and SCNEC [57], hence lacks specificity. The majority (60–90%) of ACC reveal a diagnostic fusion involving *MYB/MYBL1* with *NFIB* genes, with *MYB::NFIB* the most common [58]. Molecular testing is not required routinely, but may be performed to establish an ACC diagnosis in challenging cases.

### SWI/SNF Complex-Deficient Carcinomas (SMARCB1 & SMARCA4)

The differential diagnoses of NEC have expanded to include SWI/SNF complex-deficient sinonasal carcinomas, whether *SMARCB1* or *SMARCA4*. These tumors predominantly affect adult males [59], typically arise in the paranasal sinuses (particularly the ethmoids) [59, 60], and frequently present at an advanced stage [59]. Both are high-grade malignancies histologically characterized by a monotonous population of undifferentiated cells. Similar to NEC, tumors are cellular and composed of islands and sheets of uniformly high-grade cells with brisk mitotic activity and foci of tumor necrosis.

**Fig. 7:**
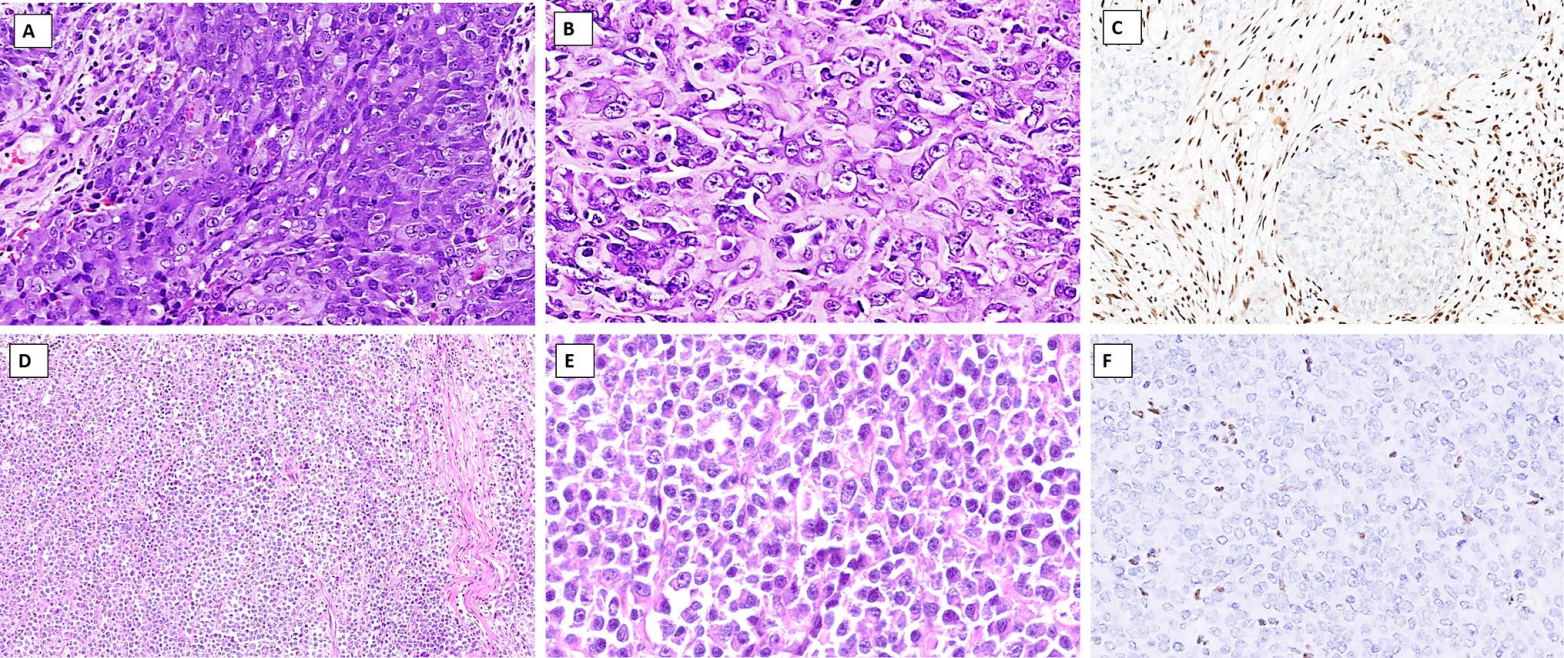
Differential diagnosis of a neuroendocrine carcinoma: SWI/SNF-deficient carcinomas. **A-C** *SMARCB1*-deficient carcinoma: Tumor shows sheets of undifferentiated carcinoma cells with basaloid morphology (**A**) and rhabdoid morphology (**B**) in a desmoplastic stroma.** C** Complete loss of SMARCB1 (INI1) immunoreactivity in the tumor nuclei with retained immunopositivity in the stromal and endothelial cell nuclei is essential for the diagnosis. **D** *SMARCA4-*deficient carcinoma showing diffuse sheets of undifferentiated cells with scanty stroma. **E** *SMARCA4-*deficient carcinoma on higher magnification reveals epithelioid tumor cells with rhabdoid morphology and conspicuous nucleoli. **F** SMARCA4 immunohistochemistry shows complete loss of SMARCA4 (BRG1) protein in the tumor nuclei, whereas the stromal cells are positive and serve as internal controls

*SMARCB1-*deficient sinonasal carcinomas predominantly exhibit a basaloid (~ 2/3) or rhabdoid (~ 1/3) morphology; the latter may be very focal (Fig. 7A, B). Additionally, sharp, punched-out vacuoles within tumor sheets, yolk sac-like morphology, and pagetoid spread along the surface epithelium may be seen in *SMARCB1*-deficient carcinomas that may aid in diagnosis when present [59].

*SMARCA4*-deficient carcinoma is composed of large, epithelioid cells lacking overt differentiation (Fig. 7D, E) [59]. Rhabdoid and basaloid cells are less frequent. The cytologic appearance mimics LCNEC, requiring exclusion of the SWI/SNF complex-deficient carcinomas.

Both entities require IHC to confirm the diagnosis. A complete loss of SMARCB1 (testing INI1]) and SMARCA4 (testing BRG1) reactivity in the tumor nuclei is essential for the diagnosis of *SMARCB1*-deficient (Fig. 7C) and *SMARCA4*-deficient sinonasal carcinoma (Fig. 7F), respectively [59]. Additionally, the tumor cells are positive for pancytokeratin (AE1/AE3, CAM5.2, OSCAR) and variably positive with CK7. Further, there is frequently reactivity with CK5/6, p63, and p40 in *SMARCB1*-deficient carcinoma, while these markers are generally negative in *SMARCA4*-deficient carcinomas. Tumor cells are negative with NUT and there is no HPV or Epstein Barr virus association [59]. It is noteworthy that both tumor types can focally express neuroendocrine markers (synaptophysin, chromogranin, and INSM1) in most *SMARCA4*-deficient carcinomas and up to 18% of *SMARCB1*-deficient carcinomas [61, 62]. Thus, INI1 and/or BRG1 must be included in a panel of immunohistochemistry studies when evaluating poorly or undifferentiated carcinomas of the sinonasal tract. Immunohistochemistry is generally sufficient for diagnosis, although FISH or sequencing can be performed to demonstrate biallelic (homozygous) deletions of the *SMARCB1* gene [59] or loss-of-function (mostly truncating) mutations in *SMARCA4*-deficient carcinomas [63].

### Tumors with Neuroectodermal Differentiation

**Fig. 8:**
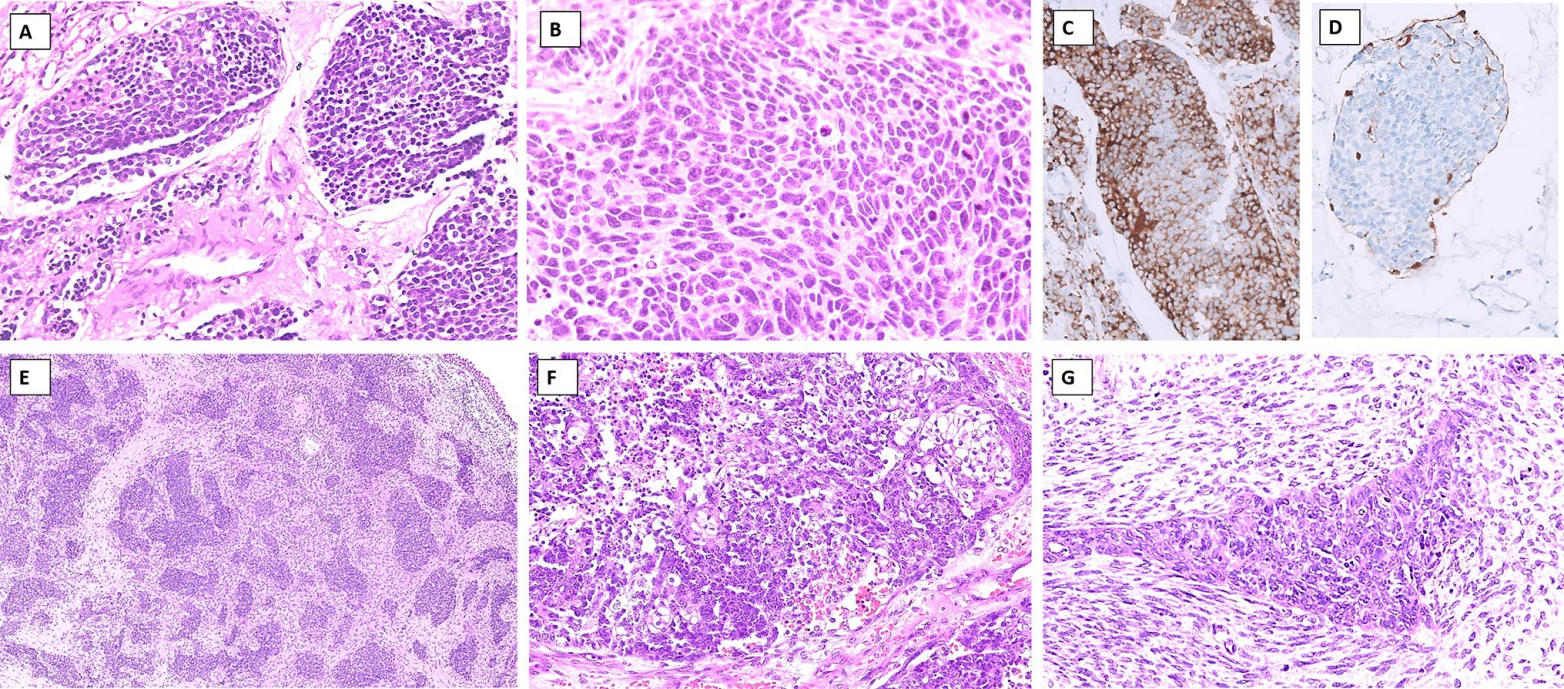
Differential diagnosis of a neuroendocrine carcinoma: Tumors with neuroectodermal differentiation. **A**–**D** Olfactory neuroblastoma (ONB). **A** Lobules of uniform cells with stippled chromatin separated by fibrovascular stroma in a Hyams low-grade ONB. **B** Loss of lobular architecture, increasing pleomorphism, and mitotic activity is seen in Hyams high-grade ONB. **C** Chromogranin is strongly positive in the tumor cells. **D** S100-positive sustentacular cells are typically seen rimming the periphery of lobules in ONB. **E**–**G** Teratocarcinosarcoma (TCS). **E** Irregular lobules of blue round cells in a variably cellular stroma is a frequent feature of TCS. **F** Nests of neoplastic cells comprising round primitive neuroectodermal cells intermixed with fetal (clear)-like squamous cells. **G** Malignant epithelial and sarcomatous components of TCS

*Olfactory neuroblastoma (ONB)* is a neuroectodermal neoplasm typically arising in the olfactory epithelium centered on the cribriform plate of the ethmoid sinus, composed of lobules of small round cells surrounded by sustentacular cells in a loose fibrovascular stroma. The morphological spectrum of ONB spans from the well-differentiated end (wherein the neoplastic cells display lobular architecture, uniform cells with stippled chromatin, rosettes and/or neurofibrillary stroma, low mitoses, and absence of tumor necrosis) (Fig. 8A) to the poorly differentiated end (which is characterized by limited lobular architecture, pleomorphism, increased mitoses, karyorrhexis, and tumor necrosis) (Fig. 8B). These features of diminishing differentiation are assembled into the Hyams tumor grades [64]. Neurons, melanin pigment, or divergent differentiation (glandular, squamous, or rhabdomyoblastic) may be seen [65–67]. A distinction of high-grade ONB from NEC is challenging and requires additional testing. ONB expresses diffuse neuroendocrine markers (synaptophysin, chromogranin, INSM1), neurofilament, and calretinin (Fig. 8C); about a third may show focal keratin reactivity [68]. The peripheral rim of sustentacular cells is highlighted by S100 protein and/or SOX10 (Fig. 8D). Recently, SATB2 and focal GATA3 expression have been demonstrated in grade 1 to 3 ONBs [69]. Tumor cells are negative for CD99, Fli1, NUT, and EBER, while INI1 and BRG1 are retained (intact). In contrast to ONB, NEC is negative for SOX10, S100, calretinin, SATB2, and GATA3 [69].

*Teratocarcinosarcoma* (TCS) is a unique sinonasal tumor that is composed of a triad of epithelial, mesenchymal, and primitive neuroectodermal components; the three elements are intermixed and any constituent may predominate in a case. A biopsy with a preponderant primitive neuroectodermal component may be mistaken for NEC if the intimately admixed epithelial or mesenchymal components are either sparse or overlooked. Fetal-like (clear) squamous epithelium, immature mesenchyme, benign and/or carcinomatous epithelium, strap cells, or sarcomatous stroma are features that suggest a diagnosis of TCS (Fig. 8E–G). In contrast to a more uniform histological picture, the varied components of TCS render a very heterogeneous low-power appearance that may serve as an important clue to the diagnosis. Immunohistochemistry can highlight the presence of epithelial and sarcomatous (especially, rhabdomyosarcomatous elements positive for desmin, MyoD1, or Myogenin) apart from neuroendocrine marker expression in the primitive neuroectodermal component. SMARCA4 (BRG1) loss, complete or partial, is identified in up to 80% of the cases [70], while a subset may reveal nuclear ß-catenin immunoreactivity [71].

### Mucosal Melanoma

Sometimes referred to as “the great mimicker,” melanoma presents with various morphological appearances and is thus a frequent tumor in many differential categories. Derived from melanocytes of the skin or mucosal linings, up to 25% of melanomas present in the head and neck region, with the scalp and cheek the two most common sites [72], while oral cavity and sinonasal tract may also be primary sites. Melanoma is usually identifiable using IHC targeting S100 protein, SOX10, MART-1 (Melan A), MITF1, and HMB45. From an embryonic perspective, melanocytes and most neuroendocrine cells both derive from the neural crest, and it is therefore not surprising to find expressional evidence of neuroendocrine differentiation in small subsets of melanoma [73, 74]. Indeed, in a retrospective study of > 300 melanomas, immunoreactivity for chromogranin A and synaptophysin was found in 2% and 8.6% of cases, respectively [75]. Focal or faint expression of at least one of these markers was observed in 37.2% of the tumor cohort, thereby highlighting the need for a careful approach when assessing neuroendocrine markers in melanocytic lesions.

### Round Cell Sarcomas

#### Ewing Sarcoma

**Fig. 9:**
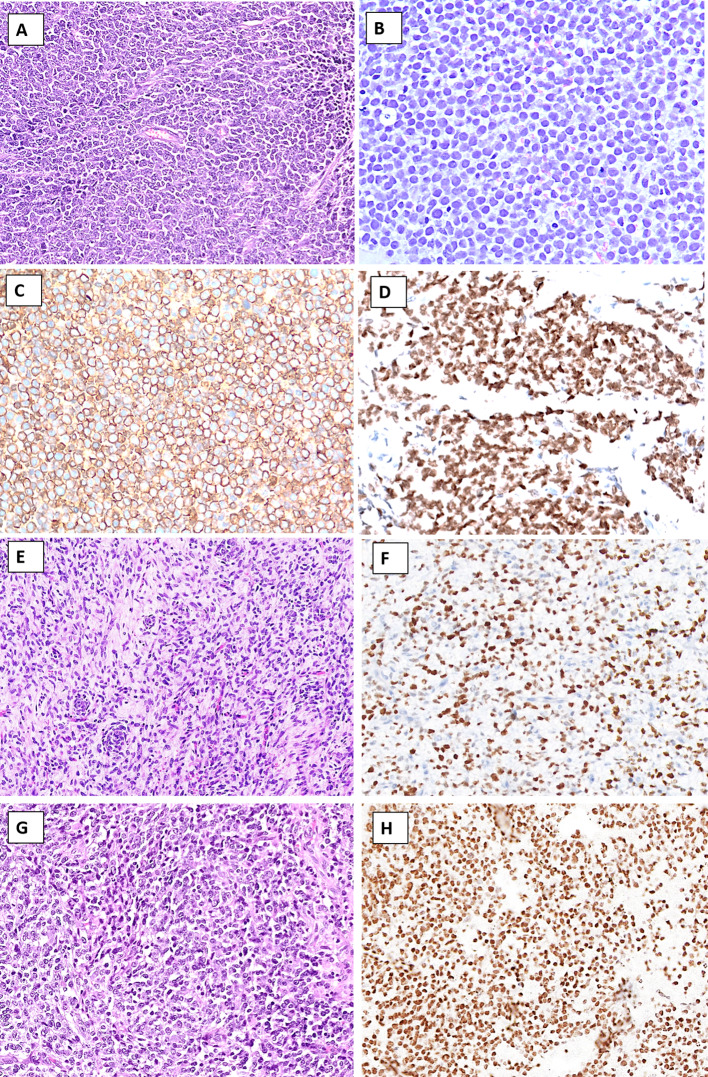
Differential diagnosis of a neuroendocrine carcinoma: Small round cell tumors. **A**–**D** Ewing sarcoma (ES). **A** Typical blue tumor appearance at low power in ES. **B** Diffuse sheets of monotonous round cells with primitive nuclei and scanty cytoplasm on high power in ES. **C** Strong and diffuse membranous staining with CD99 is typical of ES; many non-ES tumors also display CD99 positivity albeit usually focal or cytoplasmic. **D** Nuclear reactivity with NKX2.2 is characteristic although not specific of ES. **E**, **F** BCOR-related sarcoma. **E** Round cells often mixed with focal spindle cells, pale nuclear chromatin, inconspicuous nucleoli, and abundant myxoid stroma with delicate vascularity are seen in BCOR-related sarcoma. F Nuclear immunostaining for BCOR is characteristic. **G**, **H** CIC-related sarcoma. G Lobulated growth, undifferentiated round cells with mild pleomorphism, vesicular chromatin and prominent nucleoli, and delicate fibrous septae are features of CIC-related sarcomas. **H** CIC-related sarcomas display diffuse and strong nuclear WT1 immunoreactivity

Ewing sarcoma (ES) is a primitive small round cell tumor that frequently needs to be distinguished from SCNEC. ES is defined by reciprocal translocations between the *FET* (encompassing *EWSR1,* *FUS, and TAF15* genes) and the *ETS* (commonly including *FLI1, ERG, ETV1, ETV4, or FEV*) family of genes [76, 77]. Like SCNEC, it is composed of cellular sheets of monotonous small round cells, 1–2 times the size of lymphocytes, with scant cytoplasm, round to oval nuclei, delicate stippled chromatin, and devoid of conspicuous nucleoli; occasional rosettes are identified (Fig. 9A, B) [78]. Immunohistochemistry can aid in distinguishing ES from SCNEC, in which ES shows diffuse membranous positivity for CD99 (Fig. 9C) and concurrent nuclear reactivity for NKX2.2 (Fig. 9D) [79–82]. Importantly, NKX2.2 can also be seen in SCNECs [76, 77] as can CD99, but the latter is strong and membranous in ES [83]. Importantly, neuroendocrine marker positivity may be observed in ~ 50% of ES cases and ~ 30% of cases show cytokeratin expression [84, 85]. Fli1 [86] and ERG [87] reactivity are seen in cases with the respective fusions. A subtype of ES, adamantinoma-like Ewing sarcoma (ALES) tends to show a nested/lobular architecture, peripheral palisading, hyalinized stroma, and abrupt squamous differentiation; IHC evidence of squamous differentiation in the form of diffuse cytokeratin and p40/p63 reactivity is noted along with CD99 and NKX2.2 and most commonly the *EWSR1::FLI1* fusion [88, 89].

#### Other Undifferentiated Round Cell Sarcomas

Rarely, undifferentiated round cell sarcomas other than ES may be encountered that need to be distinguished from SCNEC. These include (1) round cell sarcomas with *EWSR1-*non-*ETS* fusions [90–92]; (2) *CIC*-rearranged sarcomas [93, 94]; and (3) *BCOR*-rearranged sarcomas [95, 96].

##### Round Cell Sarcomas with EWSR1–non-ETS Fusions

These are round and spindle cell sarcomas with *EWSR1* or *FUS* fusions involving partners unrelated to the *ETS* gene family. These mainly comprise *EWSR1::NFATC2* and *FUS::NFATC2* sarcomas and *EWSR1::PATZ1* sarcomas [90–92, 97–99]. Unlike conventional ES, these tumors exhibit atypical morphological features in the form of scattered enlarged cells, prominent nucleoli, or unusual clinical profiles (older patients). Nonetheless, there is considerable overlap with ES, including membranous CD99 staining. Although the pathologic spectrum is wide, some phenotypic clues to underlying genotypes can be helpful. Sarcomas with *NFATC2* fusions tend to exhibit epithelioid features [90, 100, 101], while *PATZ1* sarcomas are composed of largely undifferentiated round to ovoid neoplastic cells in a frequently sclerotic background [91, 98, 99, 102]. *NFATC2* sarcomas express diffuse CD99 (like ES) in about 50% of cases; NKX2.2, dot-like keratin, and PAX7 positivity may also be observed [103, 104]. *PATZ1* sarcomas do not consistently express CD99, however, may variably express CD34, and show a divergent phenotype with both myogenic (desmin, myogenin, MyoD1) and neurogenic (S100 protein, SOX10) markers [91, 98, 99, 102], while neuroendocrine markers are usually absent. Identification of the fusion transcripts on molecular testing is the gold standard.

*CIC-rearranged sarcomas* are round cell undifferentiated sarcomas that are defined by *CIC*-related gene fusions, mostly *CIC::DUX4* fusion (about 95%) [93, 94]. *CIC* sarcomas are composed of undifferentiated round cells, however, tend to show lobulated growth (at least focally), and delicate fibrous septae; cells display mild pleomorphism and possess vesicular chromatin and prominent nucleoli (Fig. 9G). At times, epithelioid morphology can predominate [105]. By IHC, WT1 (90–95%) (Fig. 9H) and ETV4 (95–100%) are positive and are extremely useful markers [106–108]. CD99 is positive albeit patchy and cytoplasmic [105], rather than membranous. However, NKX2.2 is typically negative [109]. Sarcomas with *CIC::NUTM1* fusions are positive for NUT protein [110, 111]. Molecular testing reveals *CIC-*related fusions.

*BCOR-related sarcoma* is a primitive round cell sarcoma showing *BCOR* genetic alterations. These tumors typically affect children with > 90% of patients being < 20 years [95]. Histology typically reveals vague nesting, round cells often admixed with focal spindled cells, pale nuclear chromatin, inconspicuous nucleoli, and abundant myxoid stroma with delicate vascularity (Fig. 9E) [95, 96]. By IHC, tumor cells show diffuse, strong BCOR (Fig. 9F), SATB2, and cyclin D1 positivity. CD99 is seen in about 50% of cases [95], but neuroendocrine markers are usually absent.

#### Alveolar Rhabdomyosarcoma (Solid Subtype)

**Fig. 10:**
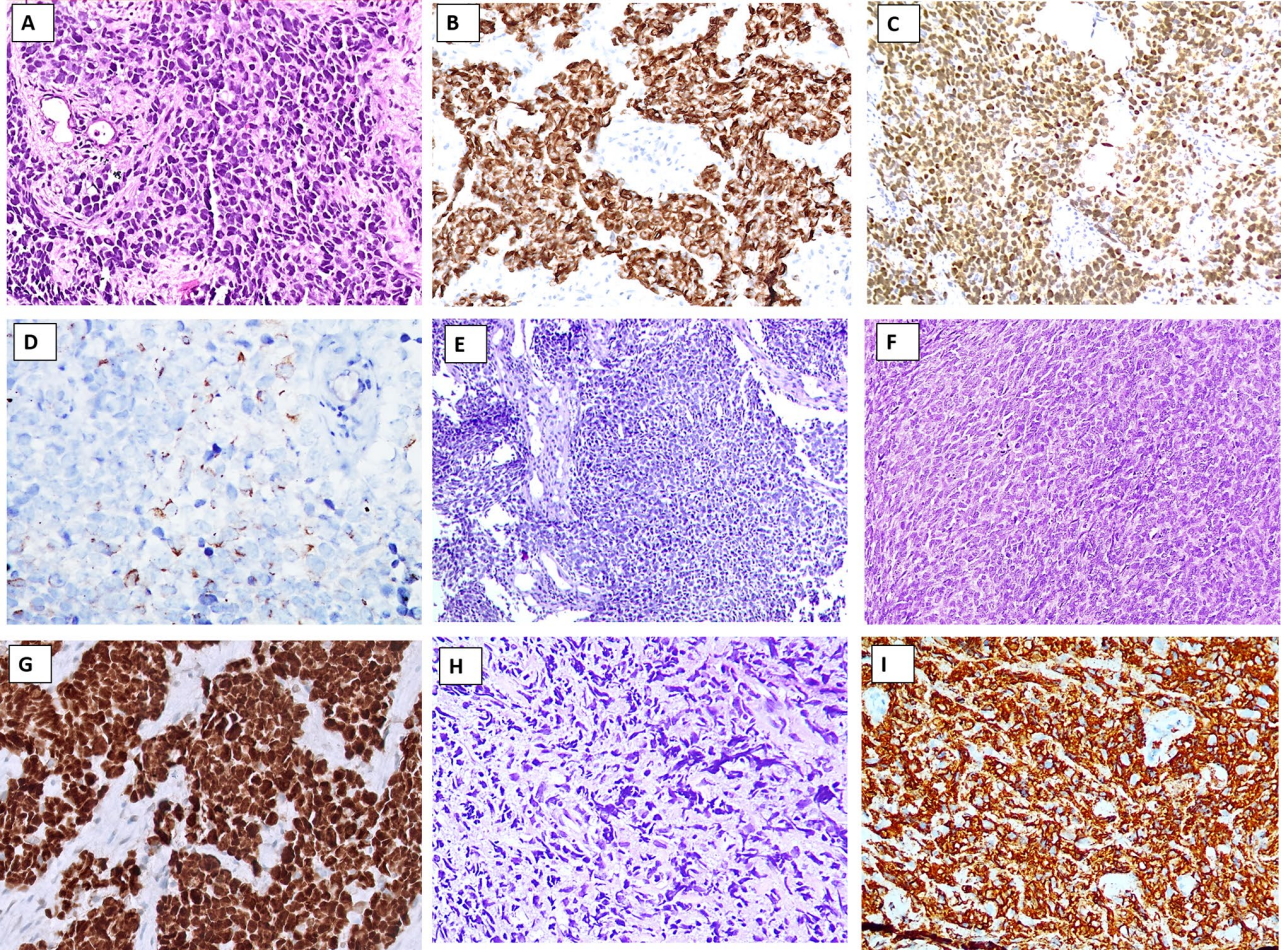
Differential diagnosis of a neuroendocrine carcinoma: Small blue round cell tumors. **A**–**D** Solid alveolar rhabdomyosarcoma (ARMS). **A** Solid sheets and islands of primitive cells with hyperchromatic nuclei, and devoid of a conspicuous alveolar pattern in solid ARMS. Tumor cells are positive for desmin (**B**) and diffusely positive for Myogenin (**C**) indicating skeletal muscle differentiation.** D** Focal expression of chromogranin is seen in ARMS in a subset of cases. This is a potentially serious caveat that can lead to an erroneous diagnosis of NEC. **E**–**G** Synovial sarcoma (SS). Blue tumor appearance of SS is typical at low power. Tumor is composed of highly cellular sheets of ovoid cells with hyperchromatic nuclei and often displays hemangiopericytoma-like vascularity. **F** Poorly differentiated SS is cellular blue tumor with increased pleomorphism and mitotic activity. **G** Diffuse and strong nuclear expression of TLE1 are seen in nearly all SS, however, is not specific; TLE1 should always be included in a panel of immunohistochemical markers. **H**, **I** Lymphoma. **H** Crushed blue tumor cells with round cell appearance seen within fibrotic stroma in a case of diffuse large B-cell lymphoma. **I** B-lineage marker, CD20 is positive on the lymphoma cells

Rhabdomyosarcoma (RMS) is a malignant mesenchymal tumor composed of primitive cells exhibiting skeletal muscle differentiation. Head and neck RMS account for about 35–40% of all RMS cases [112]. It encompasses embryonal, alveolar, pleomorphic, and spindle/sclerosing subtypes. Among the subtypes, alveolar rhabdomyosarcoma (ARMS), particularly the solid subtype, most closely mimics SCNEC [113]. In comparison to SCNEC, the patients of ARMS are much younger, with the peak age of ARMS being 10–25-year-old young adults [114–116], although cases in adults > 45 years in the sinonasal tract especially are not uncommon [113, 117, 118]. Microscopically, ARMS is characterized by cellular nests of small round cells separated by fibrovascular septae. Toward the center, the tumor cells tend to be dyscohesive conferring an alveolar configuration to the tumor; the latter is a vital diagnostic clue in favor of ARMS. In contrast, the solid subtype of ARMS is composed of diffuse sheets and lacks this nested/alveolar pattern and fibrovascular septae making it morphologically indistinguishable from NEC and small blue round cell tumors (Fig. 10A) [113, 114, 119, 120].

By IHC, cytoplasmic desmin (Fig. 10B), diffuse nuclear myogenin (Fig. 10C), and focal nuclear MyoD1 positivity are diagnostic of ARMS. Notably, neuroendocrine markers and keratins can be expressed in some cases of RMS [113]. Specific neuroendocrine markers (chromogranin A and/or synaptophysin) can be seen in up to 43% of cases (Fig. 10D) [121]. About 32% of cases can express both cytokeratins and NE markers [113, 121, 122]. This aberrant keratin and neuroendocrine marker expression in RMS can lead to an erroneous diagnosis of NEC if skeletal muscle markers are not employed. Hence, a panel of markers is essential to avoid diagnostic pitfalls. Molecular testing for ARMS diagnosis and prognostication is recommended although not necessary for distinguishing ARMS from NEC. The majority (~ 70–90%) of ARMSs contain *PAX3::FOXO1* fusions with the remaining tumors generally *PAX7::FOXO1* [114, 123].

#### Synovial Sarcoma Poorly Differentiated

Synovial sarcoma (SS) is a soft tissue sarcoma showing variable epithelial differentiation and is characterized by *SS18::SSX1, SSX2*, or *SSX4* fusions [124]. Although SS can occur at any age, the majority of patients are adolescents or young adults and < 2% of patients are older than 50 years at diagnosis [125]. Histologically, SS are cellular monophasic or biphasic tumors composed of dense sheets or vague fascicles of uniform appearing small spindle cells with ovoid, hyperchromatic nuclei with regular granular chromatin and inconspicuous nucleoli, and scant cytoplasm (Fig. 10E, F). A variable proportion of epithelial cells are seen intermixed with spindle components in the biphasic SS [126], yielding a marbled appearance on low power. The high cellularity and monomorphic appearance frequently place SS in the list of small round cell tumors, especially in limited biopsy material. The poorly differentiated subtype of SS particularly needs distinction from SCNEC. Poorly differentiated SS exhibits areas of increased cellularity, greater nuclear pleomorphism, and a high mitotic rate (> 10 mitoses per 2 mm^2^) (Fig. 10F) [127]. The cells may be spindle to round. The tumors with predominantly round cell morphology especially necessitate segregation from SCNEC [128]. Poorly differentiated tumors also tend to be more common in elderly patients [129]. By IHC, SS shows strong diffuse nuclear positivity for TLE1 in nearly all the cases (Fig. 10G), with variable positivity for CD99 and bcl2, with focal positivity for cytokeratin. Rare reports of neuroendocrine markers (synaptophysin, chromogranin A, and nestin) in FISH-confirmed SS have been reported [130]. Recently, newer antibodies, *SS18::SSX* fusion-specific antibody (E9X9V, reactive against the breakpoint) and the *SSX*-specific antibody (E5A2C, reactive against the SSX C-terminus) have shown strong diffuse nuclear staining with excellent sensitivity and specificity (> 95%) for SS [131].

### Hematolymphoid Malignancies

Lymphoma is a universal differential diagnosis for all small blue round cell tumors. High-grade diffuse large cell lymphomas, B-cell or T-cell lineage, show diffuse sheets of neoplastic cells with high nuclear-to-cytoplasmic ratio, loose chromatin, conspicuous nucleoli, and scanty cytoplasm (Fig. 10H). Brisk mitoses and apoptotic bodies are frequent. Crushing artifacts are common. Convoluted nuclei, nuclear folds, and grooves are commonly seen in T-cell lineage tumors. Tumor cells infiltrating through fibrotic stroma may simulate clustering similar to carcinomas. Due to overlapping features, distinction from NEC is usually required, especially in a limited biopsy. Immunohistochemistry can readily help in segregating lymphomas from NEC. Lymphomas are positive for hematolymphoid markers including CD45RB, while negative with pancytokeratins and neuroendocrine markers (Fig. 10I).

## Conclusion

Pathology is often considered a specialty in which experience is measured in case volume or years of practice—and given the rarity of neuroendocrine neoplasms and their mimics, diagnosticians outside of tertiary diagnostic centers may have difficulties in acquiring adequate experience for some of these entities. Moreover, identification of these lesions in the head and neck region usually requires an integration of clinical information, imaging findings, histomorphology, and immunohistochemical assessments and not all hospital settings may provide the latest antibody panels or molecular platforms to assess some of the key features. Even so, when faced with a head and neck tumor in which NEC is a potential differential diagnosis, careful exclusion of the top ten mimickers as highlighted herein will facilitate narrowing the diagnoses to the correct classification.

## Data Availability

Not applicable.
